# Commentary: Central-acting therapeutics alleviate respiratory weakness caused by heart failure–induced ventilatory overdrive

**DOI:** 10.3389/fphys.2018.00554

**Published:** 2018-05-23

**Authors:** Amy M. Pastva, Julia K. L. Walker

**Affiliations:** ^1^Duke University School of Medicine, Duke University, Durham, NC, United States; ^2^Duke University School of Nursing, Duke University, Durham, NC, United States

**Keywords:** respiratory muscle weakness, exercise training, heterodimer receptor, blood-brain barrier, ventilatory overdrive, angiotensin drive to breathe, GPCR signaling

Heart failure (HF) is a chronic, progressive condition that manifests not only in cardiac dysfunction but also in respiratory dysfunction. Diaphragmatic myopathy is common and contributes to dyspnea and exercise intolerance in advancing stages of HF (reviewed in Cahalin and Arena, 2015; Dubé et al., 2016). The potential pathophysiological mechanisms driving diaphragmatic myopathy over the evolution of HF have remained elusive. Long-held views suggest that a HF-induced increase in lung mechanical load (either from pulmonary edema or lung fibrosis) is the primary cause of progressive diaphragmatic myopathy (Mahdyoon et al., 1989; Chomsky et al., 1997; Gheorghiade et al., 2010; Cahalin and Arena, 2015; Dubé et al., 2016). However, Foster et al. provide compelling evidence for an additional explanation (Foster et al., 2017). In the article “Central-acting therapeutics alleviates inspiratory weakness caused by HF-induced ventilatory overdrive” they elegantly demonstrate that initiation of diaphragmatic myopathy is mediated by a hormonal mechanism independent of lung mechanical load. They used two mouse models of pressure-overload-induced HF to show that activation of functionally codependent angiotensin type-1 (AT_1_) receptors and beta-adrenergic receptors (β-ARs) triggers an excessive central drive to breathe that underlies the development of diaphragm myopathy. This ventilatory (AbdAlla et al., 2005) overdrive was associated with increased mRNA expression of PERK (protein kinase R–like endoplasmic reticulum kinase), hyperphosphorylation-mediated inhibition of EIF2a (eukaryotic translation initiation factor 2α) and consequent reduction in protein translation and cross-sectional area of the diaphragm. Given that only blood-brain-barrier (BBB)-permeant antagonists of the AT_1_ receptors and β-ARs were able to diminish diaphragmatic myopathy, Foster et al. concluded that receptors behind the BBB were responsible for ventilatory overdrive. These atrophic changes preceded detectible evidence of diaphragm force changes and weakness. Thus, ventilatory overdrive associated with HF may now be thought of, and perhaps treated, as an early hormonal complication of the disease.

In our opinion, the important therapeutic impact of the Foster et al., findings is twofold. First, their study provides rationale for early initiation of treatment to prevent ventilatory overdrive—perhaps upon diagnosis of HF, but certainly well before lung structural or mechanical changes occur. Second, this work highlights the therapeutic importance of considering the BBB-permeant and other characteristics of AT_1_ receptor and βAR blockers used to treat HF. Before commenting on the pharmacological aspects of the Foster manuscript it may be instructive to provide clarification of some of the complex terminology and concepts used in the manuscript.

## Ventilatory drive and overdrive

The drive to breathe is reflected by the frequency of action potentials that originate at the medullary respiratory center and are transmitted to the diaphragm via the phrenic nerve (Whitelaw et al., 1975). This drive to breathe is influenced by numerous types of sensory information, not the least of which is chemical. The main finding in the Foster et al. paper is that blockade of hormones typically elevated in HF significantly mitigates the increased drive to breathe and diaphragmatic weakening observed in their murine heart disease model.

Foster et al. use the term “ventilatory drive” to describe the drive to breathe but there are several other synonyms including central drive, neural drive, and respiratory drive. Because measurement of phrenic nerve activity, the most direct measure of ventilatory drive, is not feasible in humans and difficult in animals, minute ventilation can be used as a surrogate. However, in pathological conditions minute ventilation may not accurately reflect the drive to breathe (Celli et al., 1997). Thus, in disease states the ventilatory drive to the diaphragm can be inferred from the magnitude of respiratory pressure generated by the contracting muscle. In humans the pressure generated during the first 0.1 sec of a very brief inspiratory occlusion (P_0.1_) during a normal tidal breath reflects the ventilatory drive (Celli et al., 1997). In the Foster study ventilatory drive is approximated by the inspiratory pressure (P_I_), which was calculated from esophageal pressure measured during normal eupneic breathing in the anesthetized mouse.

Elevated mouth occlusion pressure (P_0.1_) or inspiratory pressure (P_I_) reflects increased activation of the phrenic nerve and concomitant increased contraction of the diaphragm.

Diaphragmatic weakness is assessed in humans by voluntarily generating a maximal inspiratory pressure (P_Imax_) (Celli et al., 1997; Lin and Lin, 2012). Since a volitional maneuver is not feasible in mice, Foster et al. measured esophageal pressure during a 25 s tracheal occlusion in anesthetized mice (P_IOOC_). In summary, inspiratory pressure is directly proportional to ventilatory drive, maximal inspiratory pressure is inversely proportional to diaphragm weakness and, although the mechanisms are not well understood, chronic elevated ventilatory drive is associated with diaphragmatic weakness.

Foster et al. use the term “ventilatory overdrive” and define it as a persistent increase in ventilatory drive triggering diaphragmatic myopathy. This is a somewhat cumbersome definition, but deconstructed it essentially reflects the combination of both an increase in P_I_ and a decrease in P_IOOC_. The utility of this somewhat confusing term becomes apparent in the manuscript discussion where the focus rests on the effect of hormones to promote progressive diaphragmatic weakening associated with an increased drive to breathe.

## Early RAAS-targeted interventions to combat diaphragmatic myopathy

Although HF is a heterogeneous condition with multiple etiologies, all etiologies lead to chronic activation of the renin-angiotensin-aldosterone system (RAAS) and the sympathetic nervous system (McMurray et al., 2014; McMurray, 2015; Metra and Teerlink, 2017). The RAAS consists of a two-arm axis: an excitatory angiotensin II (ANGII)/AT_1_R/ACE (angiotensin converting enzyme) arm and a protective AT2R/ACE2 arm. Increasing evidence suggests that in HF there is an imbalance to favor the excitatory arm, leading to sympathoadrenergic activation and that this imbalance is modulated in part in the cardiovascular-control regions of the brain (reviewed in Zucker et al., 2014). Therefore, the use of BBB permeant interventions that restore RAAS balance and/or cause sympathoinhibition are plausible therapeutic strategies. Certainly, the data in Foster et al. challenge us to consider the potential application of BBB permeant pharmacological interventions, especially early in the HF disease process, to inhibit AT_1_ receptor- and βAR-stimulated ventilatory overdrive and mitigate diaphragmatic myopathy.

Nonpharmacological interventions such as physical exercise may also be important to consider as an early therapeutic strategy given its positive effects on mortality, morbidity, functional capacity, and quality of life in HF (De Maeyer et al., 2013). For instance, aerobic exercise has been shown to dampen RAAS and sympathoadrenergic activation and reduce the typically elevated circulating levels of ANGII and catecholamines characteristic of HF (Coats et al., 1992; Braith et al., 1999; Passino et al., 2006a,b; Gielen et al., 2010). Additionally, exercise has also been shown to dampen central RAAS activation (reviewed in Zucker et al., 2014). Thus, exercise may be an important therapeutic approach to mitigate respiratory dysfunction in HF via its inhibition of RAAS and catecholamines. Targeting the respiratory muscles themselves may also be beneficial given that inspiratory muscle strength is correlated with exercise capacity and peak oxygen consumption and is an independent predictor of survival (Meyer et al., 2001). For example, inspiratory muscle training using a threshold device, examined across multiple systematic reviews, has been shown to improve one or more pathophysiological manifestations of HF such as dyspnea, maximal inspiratory and expiratory pressures, respiratory muscle strength, muscle sympathetic activity, and exercise capacity (reviewed in Cahalin and Arena, 2015). Slow breathing exercises in patients with HF aims to reduce respiratory rate and improve dyspnea and exercise tolerance. It has been demonstrated to decrease chemoreflex activity to hypoxia and hypercapnia and increase baroreflex activity through improved vagal tone (Bernardi et al., 1998, 2002; Parati et al., 2008). Thus, centrally-targeted AT_1_ and βAR receptor modulating pharmaceuticals in combination with physical exercise early in HF progression may combat ventilatory overdrive and mitigate respiratory muscle myopathy to improve HF symptomatology, exercise tolerance, and potentially severity classification.

## ANGII drive to breathe and the blood brain barrier

The notion that ANGII regulates ventilation is not new. Potter and McCloskey (1979) and Alexander and Lumbers (1981) showed that ANGII stimulates the drive to breathe in anesthetized dogs. Subsequent studies showed this to also be true in dogs that were conscious—where arterial baroreflexes, not dampened by anesthesia, can inhibit ventilation (Ohtake and Jennings, 1993). When the blood pressure-raising effects of infusion of ANGII (a vasoconstrictor) were normalized by concomitant infusion of SNP (a vasodilator), the ANGII-mediated drive to breathe was revealed to be quite pronounced (Ohtake et al., 1993). If this translates to humans, then pathologically elevated levels of ANGII may indeed cause ventilatory overdrive.

However, Foster et al., did not measure systemic or brain levels of ANGII, nor did they speculate about the specific central location at which ANGII acts to stimulate ventilation. In fact, they state that the ability of ANGII/catecholamines to cross the BBB is controversial. In conscious dogs, systemic-derived ANGII cannot cross the BBB; however, it is still able to centrally stimulate ventilation by activating circumventricular organ neurons which effectively lack a BBB but project to medullary cardiorespiratory control centers behind the BBB (Walker and Jennings, 1994). Because only BBB-permeant antagonists of the AT_1_ receptor and βAR rescued the mice, Foster et al. concluded that receptors behind the BBB mediate ventilatory overdrive. An alternative explanation is possible in that the same molecular characteristics that enable an antagonist to cross the BBB may also allow it to regulate cell signaling downstream of the AT_1_ receptor/βAR heterodimer in a unique way. If true, the BBB-permeant antagonists may have acted at circumventricular organ neurons, rather than those behind the BBB, to exert an effect on ventilatory drive. Yet another possibility is that the chronic disease model [transverse aortic constriction (TAC)] employed in this study may have initiated changes in the heterodimer ratio of AT_1_ receptors to βARs that resulted in signaling changes at the diaphragm muscle cells themselves, or myopathy secondary to activation of ventilatory overdrive.

## G protein-coupled receptor theory

βAR and AT_1_ receptor are two very well characterized G protein-coupled receptors (GPCRs). GPCRs, the largest family of cell surface receptors, are implicated in numerous diseases and thus their signaling pathways are prime targets for therapeutic intervention (Lefkowitz, 2004). Intra- and inter-molecular electrostatic forces determine the 3-dimensional shape of a receptor and thus, its function (Matthew, 1985). The current GPCR paradigm states that signaling is transduced by both a G protein-dependent and a G protein-independent/βarrestin-dependent signaling pathway (Lefkowitz and Shenoy, 2005). For example, orthosteric binding of a ligand causes the receptor to shift into a shape which triggers G protein- and/or βarrestin-dependent signaling (Lefkowitz, 2004). Moreover, each receptor ligand, based on its molecular structure, can activate these two signaling pathways with differing efficacies resulting in biased signaling of one pathway over the other. Also, exogenous drugs, endogenous molecules and ions that electrostatically interact with receptor regions (other than the binding pocket) act as allosteric modulators or receptor signaling. For example, Zn2+ enhances agonist affinity and second messenger signaling downstream of the β2AR (beta-2-adrenergic receptor) in membranes prepared from Sf9 cells (Swaminath et al., 2002).

Adding complexity to GPCR signaling mechanisms, we now know that two or more molecularly dissimilar and individually functional GPCRs can combine to form a receptor signaling complex that has a distinct pharmacology (Angers et al., 2002; Smith and Milligan, 2010; Rivero-Müller et al., 2013). GPCRs were typically thought of as monomers capable of signaling completely independently. Although the majority of receptors within a specific GPCR type do indeed function as monomers, a functionally relevant proportion of them can dimerize (Whorton et al., 2007). In 2003 Barki-Harrington et al. (2003) were the first to show that AT_1_ receptor and βAR heterodimerize. Moreover, they demonstrated that the tachycardic effect of isoproterenol was inhibited by either propranolol or valsartan in mice. Since publication of that seminal finding, numerous other investigators have described receptor heterodimerization (Barki-Harrington et al., 2003; Noma et al., 2007; Tilley, 2011; Siddiquee et al., 2013; Wilson et al., 2013). The electrostatic interactions derived from the close proximity of receptors within the heterodimer complex impacts receptor conformation and thus, signaling (Kenakin and Miller, 2010). Moreover, pathway intermediates downstream of one receptor can exert lateral allosterism on the other receptor in a heterodimer (Wilson et al., 2013). Taken together, receptor heterodimers are allosteric machines where activation of a receptor in its monomer form does not necessarily yield the same physiological outcome as its activation when complexed as a heterodimer (Goupil et al., 2013). Thus, alterations in the ratio of monomer to heterodimer may occur during times of stress and lead to pathogenesis. For example, angiotensin and bradykinin 2 receptor heterodimers sensitize vascular smooth muscle cells to the pro-contractile effects of ANGII (AbdAlla et al., 2000). This has been shown to contribute to experimental hypertension (AbdAlla et al., 2005) and human preeclampsia (AbdAlla et al., 2001). Additionally, the work from Siddiquee et al. (2013) concludes that loss of apelin or its receptor, which tonically inhibits AT_1_ receptor signaling as part of a heterodimer, may lead to pathogenesis in cardiovascular disease.

Although AT_1_ receptor and βAR transmodulation clearly underlies the results in the Foster et al., it is prudent to consider that biased cell signaling may also play a role through its effect on heterodimer signaling. Others have shown that [Sar^1^,Ile^4^,Ile^8^]-ANGII (SII), a βarrestin-biased angiotensin receptor agonist, inhibits/dampens bradykinin 2 receptor signaling (Wilson et al., 2013). Thus, a ligand that triggers biased signaling at one heterodimer receptor type may in turn impact signaling of the second receptor type. This type of lateral allosterism may be at play in HF since carvedilol, and not metoprolol, is a weak agonist of βarrestin-signaling,(Wisler et al., 2007) which could be contributing to ventilatory overdrive.

In summary, the current GPCR signaling paradigm suggests that a single ligand can trigger more than one signaling pathway downstream of a particular receptor (G protein and βarrestin pathways), that these signaling pathways are not necessarily equally activated (biased signaling) and that signaling at one receptor can modulate the signaling at a second receptor family (heterodimer transmodulation). Regulation of GPCR signaling is extremely complex and our current understanding of how most pharmacologic drugs, including those used in this study, impact βarrestin-dependent-, biased- and heterodimer-signaling is incomplete, as is the cell specificity of the drug effects. Moreover, how the ratio of monomer to heterodimer receptors may impact pathology (or vice-versa) is largely unexplored in the context of drug design. Although a daunting task, the design of novel molecules that have pluridimensional efficacies holds great promise for treatment of multiple morbidities associated with complex diseases such as HF and diaphragmatic myopathy.

## Conclusion

The Foster et al., study significantly advances our understanding of diaphragmatic myopathy and helps to reconcile why various drugs with comparable cardiovascular effects can have differing benefits on symptomatology and mortality and reveal potential pathways for early intervention and future translational investigations.

## Author contributions

All authors listed have made a substantial, direct and intellectual contribution to the work, and approved it for publication.

### Conflict of interest statement

The authors declare that the research was conducted in the absence of any commercial or financial relationships that could be construed as a potential conflict of interest.
